# Review article: new treatments for advanced differentiated thyroid cancers and potential mechanisms of drug resistance

**DOI:** 10.3389/fendo.2023.1176731

**Published:** 2023-06-26

**Authors:** Sarah Hamidi, Marie-Claude Hofmann, Priyanka C. Iyer, Maria E. Cabanillas, Mimi I. Hu, Naifa L. Busaidy, Ramona Dadu

**Affiliations:** Department of Endocrine Neoplasia and Hormonal Disorders, The University of Texas MD Anderson Cancer Center, Houston, TX, United States

**Keywords:** drug resistance, immunotherapy, neoadjuvant, metastatic thyroid cancer, radioiodine refractoriness, tyrosine kinase inhibitor, novel treatments

## Abstract

The treatment of advanced, radioiodine refractory, differentiated thyroid cancers (RR-DTCs) has undergone major advancements in the last decade, causing a paradigm shift in the management and prognosis of these patients. Better understanding of the molecular drivers of tumorigenesis and access to next generation sequencing of tumors have led to the development and Food and Drug Administration (FDA)-approval of numerous targeted therapies for RR-DTCs, including antiangiogenic multikinase inhibitors, and more recently, fusion-specific kinase inhibitors such as RET inhibitors and NTRK inhibitors. BRAF + MEK inhibitors have also been approved for *BRAF*-mutated solid tumors and are routinely used in RR-DTCs in many centers. However, none of the currently available treatments are curative, and most patients will ultimately show progression. Current research efforts are therefore focused on identifying resistance mechanisms to tyrosine kinase inhibitors and ways to overcome them. Various novel treatment strategies are under investigation, including immunotherapy, redifferentiation therapy, and second-generation kinase inhibitors. In this review, we will discuss currently available drugs for advanced RR-DTCs, potential mechanisms of drug resistance and future therapeutic avenues.

## Introduction

Differentiated thyroid cancers (DTCs) have an excellent prognosis in most patients, with an overall 5-year relative survival rate of 98.4% according to the SEER database (1). However, a subtype of patients, representing 5-10% of all DTCs, will develop distant metastasis, most frequently in the lungs and bones (2). Prognosis remains favorable as long as metastatic disease is radioiodine-avid (3). Yet, 50% of metastatic DTCs are refractory to radioactive iodine (RAI), which is associated with poor outcomes and a 10-year survival rate of about 10% (4). On the other hand, many patients with advanced radioiodine refractory DTC (RR-DTC) can have an indolent or slowly progressive disease for many years. Thus, as multiple advances have been made in the treatment of advanced RR-DTCs in the last decade with multiple new therapeutic options, current challenges include identifying the appropriate timing for treatment initiation as well as choice of the right therapy.

## When to treat RR-DTC

The first step in treating advanced DTCs is to properly identify radioiodine refractory disease. In fact, until disease is considered unresponsive to RAI, ^131^I remains the gold standard in the treatment of metastatic advanced disease (3). However, taking into account the toxicity associated with high cumulative doses of RAI, it is crucial to properly identify when this therapy is no longer beneficial to the patient. The definition of RR-DTC can be challenging in clinical practice and remains somehow controversial. In most publications (2–6), RAI-refractory (RAI-R) disease is defined as either: (1) absence of RAI uptake outside the thyroid bed on the first posttherapy whole body scan, (2) loss of RAI concentration in a tumor tissue which was previously proved as RAI-avid, (3) concentration of RAI in some tumor lesions but not in others, and (4) progression of metastatic disease despite significant concentration of RAI, within a relevant time frame, usually considered as 6-12 months after ^131^I therapy. A fifth criterion which is highly debated is disease progression in a patient who has received ≥ 600 millicuries (mCi) of ^131^I. This is based on a single study which showed no further complete remissions after a cumulative dose of 22.2 GBq (600 mCi) (7). Therefore, factors such as response to previous therapies, duration of response, RAI uptake on diagnostic whole-body scan as well as previous treatment toxicity and patient preference should all be taken into account when considering if additional RAI therapy is indicated, rather than cumulative dose alone. Finally, ^18^F-FDG PET/CT could also be useful in identifying RAI-R disease. For instance, a study showed that a SUVmax greater than 4.0 in ^18^F-FDG avid metastases has a sensitivity of 75.3% and a specificity of 56.7 for predicting absence of ^131^I avidity (8).

Although associated with risk of progression and poorer prognosis, not all RAI-R disease needs immediate therapy. In fact, RAI-R metastatic DTCs can have an asymptomatic and indolent clinical course for several years. Such patients can be managed with active surveillance and TSH suppression alone as long as disease is asymptomatic, there is no or minimal progression, and tumor burden is low (2–4, 9). Active surveillance includes regular cross-sectional imaging of known sites of distant disease (every 3-12 months), serum thyroglobulin (Tg) and Tg antibody measurement, and as needed ^18^FDG-PET/CT whole body imaging, especially when Tg levels are increasing without explanation from cross-sectional imaging (2, 3, 9).

During this surveillance period, various scenarios can occur. First, disease can remain stable and asymptomatic, thus requiring no further intervention. Alternatively, there can be significant progression in one single lesion putting the patient at risk of complications or symptoms. This should be managed by locoregional therapy when feasible, including external beam radiation, stereotactic radiosurgery, thermal ablation, transarterial chemoembolization, and/or surgery (2, 3, 9). Finally, when local therapies are not feasible, or there is tumor progression despite local therapy, or there is significant disease progression in multiple lesions affecting more than one organ, then systemic therapy with kinase inhibitors becomes indicated (9).

## Molecular basis of differentiated thyroid cancer

**Figure 1:**
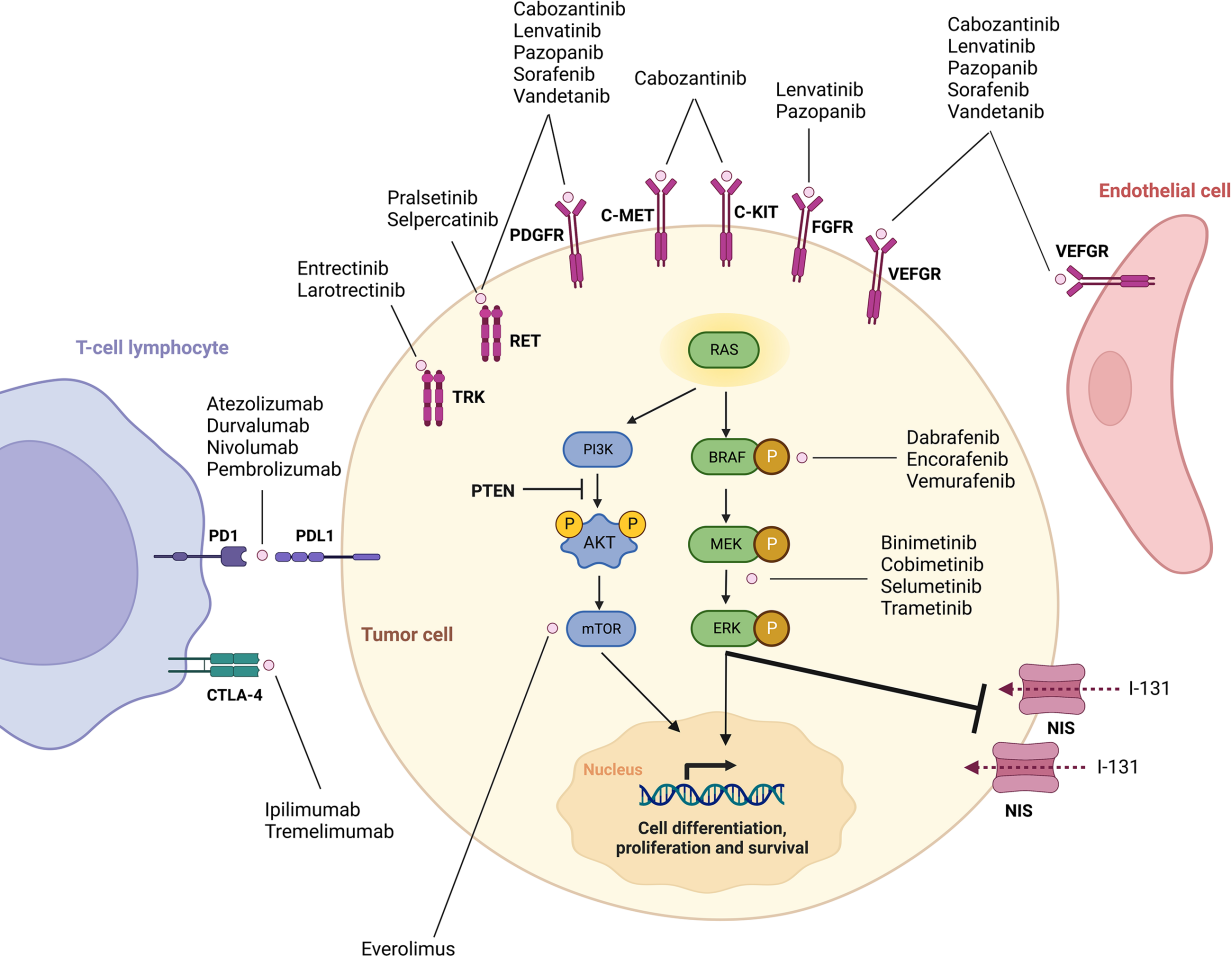
Overview of mechanisms of tumorigenesis in differentiated thyroid cancer and targets of currently available drugs. Adapted from Cabanillas et al., Targeted Therapy for Advanced Thyroid Cancer: Kinase Inhibitors and Beyod. Endocr Rev. 2019;40(6):1573-604. By permission of Oxford University Press, License# 5497811051598.

Selecting the right kinase inhibitor to treat advanced progressive RR-DTC requires a comprehensive knowledge of the genetic alterations underlying these tumors. In fact, over the last decade, better understanding of the molecular mechanisms driving DTCs and RAI refractoriness has allowed the development of multiple targeted therapeutic agents (**Figure 1**).

The mitogen-activated protein kinase (MAPK) pathway is central to the pathogenesis of papillary thyroid carcinomas (PTCs). Mutually exclusive activating somatic alterations of genes encoding effectors in this pathway were found to represent over 80% of the known genetic alterations in these tumors in The Cancer Genome Atlas (TCGA) (10). *BRAF* V600E oncogenic mutations are the most frequent, encountered in about 60% of PTCs, followed by *RAS* point mutations and *RET* fusions (10). Rearrangements involving *ALK* and *NTRK* genes encoding tyrosine kinase receptors have also been described and are of particular interest since therapies targeting these mutations are now available (5, 11, 12). When no mutation in the MAPK pathway is identified, alterations in members of the phosphoinositide 3-kinase (PI3K) pathway are usually detected, including *PTEN*, *PIK3CA* and *AKT1* mutations, although those are relatively rare (5, 10). *EIF1AX* has been described as a novel driver oncogene in approximate 1% of PTCs using the TCGA, and is mutually exclusive with MAPK mutations (10). Other mutations occasionally encountered in PTCs include fusions involving *BRAF, THADA, MET, FGFR2 and ROS1 (* 10, 13).

Mutation profile can help predict tumor behavior and RAI refractoriness (14). In fact, it has been well described that tumors driven by *BRAF* V600E mutations exhibit high MAPK-signaling output and significant reduction in the expression of genes responsible for iodine uptake and metabolism, such as the sodium-iodide symporter (NIS) (10, 11). Tumors harboring *BRAF* V600E mutations had a significantly lower differentiation score in the TCGA cohort when compared to those with *RAS* mutations (10), explaining the decreased RAI uptake and responsiveness seen in *BRAF*-mutant tumors. A mouse model developed by Chakravarty and colleagues (15), in which oncogenic expression of *BRAF* V600E in thyroid follicular cells is inducible by doxycycline administration, supports this observation. Following induction of *BRAF* V600E expression, mice developed high-grade PTCs with increased MAPK transcriptional output and impairment of thyroid-specific gene expression, including near complete loss of NIS. Nevertheless, given the high frequency of *BRAF* mutations in PTC, and the indolent course of most cases, *BRAF* V600E mutation is likely insufficient on its own to explain the aggressive behavior of some tumors. Next generation sequencing (NGS) of advanced PTCs has shown that aggressive tumor behavior and recurrence are more likely when more than one oncogenic mutation is present, especially when *TERT* promoter, *TP53, PIK3CA* and/or *AKT1* mutations co-exist with *BRAF* V600E mutations (3, 12, 16, 17). Moreover, these mutations may act in concert with *BRAF* V600E mutations to induce RAI refractoriness by leading to increased signaling in the MAPK and PI3K/AKT/mTOR pathways and further reducing NIS signaling (4, 18)

Follicular thyroid carcinomas (FTCs), which represent only 2-5% of all thyroid cancers, are most often associated with mutations involving the *RAS* oncogene, and rarely, *PAX8-PPARγ* rearrangements (11, 12). Mutations in genes encoding components of the PI3K/PTEN/AKT signaling pathway are also frequent: for instance, *PTEN* mutations are encountered in up to 10% of sporadic FTCs (19, 20). *TERT* promoter mutations can also be found in FTCs and are associated with more aggressive disease (16).

Oncocytic thyroid carcinomas (OTCs, previously Hürthle cell carcinomas), now considered as a separate subtype of thyroid cancer, harbor a mutational profile distinct from those of PTCs and FTCs (21, 22). In fact, OTCs are not associated with *BRAF* mutations, and rarely harbor *RAS* mutations or oncogenic fusions (5, 21, 22), justifying that it was inappropriate to consider them as a subtype of FTC. OTCs are rather characterized by near-haploid chromosomal content in most tumors, as well as mitochondrial DNA alterations (22). Furthermore, genes found to be more frequently altered in OTCs include *DAXX, TP53, TERT* promoter and *EIF1AX*, among others (5, 21, 22).

Agents targeting some of the mutations known to contribute to thyroid cancer pathogenesis have been developed in the last two decades, significantly changing the outcome of patients with advanced DTCs. **Table 1** summarizes all currently FDA-approved kinase inhibitors for RR-DTCs.

**Table 1:** FDA-approved Drugs in advanced RR-DTC.

Drug	Target	Number of patients with DTC in study*	Efficacy results	Reference
**Cabozantinib**	VEGFR2, AXL, MET, RET, C-KIT	125	ORR: 15% Median PFS: NR	Brose et al. (23)
**Dabrafenib** **(Single agent)**	*BRAF* V600E	26	ORR: 35% Median PFS: 10.7 months	Busaidy et al. (24)
**Dabrafenib + trametinib**	Dabrafenib: *BRAF* V600E Trametinib: MEK	27	ORR: 30% Median PFS: 15.1 months	Busaidy et al. (24)
**Entrectinib**	*NTRK* fusions, *ALK, ROS1*	13	ORR: 53.8% Median PFS: 19.9 months	Demetri et al. (25)
**Larotrectinib**	*NTRK* fusions	21	ORR: 71% 24-month PFS: 86%	Waguespack et al. (26)
**Lenvatinib**	VEGFR1-3, RET, FGFR1-4, PDGFR, KIT	261	ORR: 64.8% Median PFS: 18.3 months	Schlumberger et al. (27)
**Pralsetinib**	*RET* fusions and mutations	21	ORR: 86% Median PFS: 19.4 months	Mansfield et al. (28)
**Selpercatinib**	*RET* fusions and mutations	19	ORR: 79% 12-month PFS: 64**%**	Wirth et al. (29)
**Sorafenib**	VEGFR1-3, RET, RAF, PDGFR-β	207	ORR: 12.2% Median PFS: 10.8 months	Brose et al. (30)

NR, not reached; VEGFR1-3, VEGF receptors 1-3l FGFR1-4, FGF receptors 1-4.

*Data from the highest-phase trials were used. When more than one trial of the same phase was available, their data were pooled.

## Kinase inhibitors

### FDA-approved non-specific tyrosine kinase inhibitors

The first two agents that were approved by the Food and Drug Administration (FDA) for the treatment of patients with locally recurrent or metastatic, progressive, RR-DTC are sorafenib (approved in 2013) and lenvatinib (approved in 2015), both multikinase inhibitors (MKI) with anti-angiogenic action through inhibition of the vascular endothelial growth factor receptors (VEGF-R) 1,2 and 3. In fact, DTCs were shown to exhibit disorganized vasculature and cancer-cell hypoxia, leading to an increased activation and expression of VEGF-R and a dependence on its signaling for tumor survival (11). VEGF and its receptor are therefore interesting therapeutic targets. Sorafenib and lenvatinib also have variable inhibitory actions on other kinases, including RET, fibroblast growth factor (FGF) and platelet-derived growth factor (PDGF) receptors.

#### Sorafenib

Efficacity of sorafenib for the treatment of advanced DTC was demonstrated in the phase 3 randomized, double-blind, placebo-controlled trial DECISION (30). This study enrolled 416 patients with locally advanced or metastatic RR-DTC that had progressed in the previous 14 months and had not been previously treated with targeted therapy or chemotherapy. Median progression-free survival (PFS) was significantly longer in the sorafenib group (10.8 months) compared to the placebo group (5.8 months; hazard ratio [HR] 0.59, 95% CI 0.45-0.76; p < 0.0001). Objective response rate (ORR) and disease control rate (DCR) were also significantly higher in the sorafenib group, respectively 12.2% compared with 0.5%, and 54.1% compared with 33.8%. Overall survival (OS) did not differ significantly between the treatment groups (HR 0.80; 95% CI 0.54-1.19; p =0.14), but patients were allowed to cross over from the placebo to the treatment arm at disease progression.

#### Lenvatinib

Similarly, the phase 3 SELECT trial led to the FDA approval of lenvatinib (27). This randomized, double-blind, placebo-controlled trial included 261 patients with RR-DTC that had progression within the previous 13 months. Patients treated with up to one prior tyrosine kinase inhibitor (TKI) were included. Median progression-free survival was 14.7 months longer in the lenvatinib group (PFS 18.3 versus 3.6 months; HR 0.21, 99% CI 0.14-0.31; p < 0.001). This PFS benefit was independent of previous TKI therapy. ORR was 64.8% in the lenvatinib group as opposed to 1.5% in the placebo group (Odds ratio [OR] 28.87; 95% CI 12.46 – 66.86; p < 0.001), with four complete responses (CR). Although no significant difference in OS was observed between the two groups (HR for death 0.73, 95% CI 0.50-1.07; p=0.10), there was a significant survival benefit with the use of lenvatinib in patients over the age of 65 despite crossover from the placebo to the treatment arm at disease progression (OS not reached VS 18.4 months; HR 0.53; 95% CI 0.31-0.91; p = 0.02).

Although they led to significant prolongation of PFS, these agents were associated with adverse events (AEs) in virtually all patients, including grade ≥ 3 adverse events in 75.9% of patients on lenvatinib and 37.2% of patients on sorafenib. AEs led to discontinuation of lenvatinib in 14.2% of patients and sorafenib in 18.8%, while treatment interruptions and dose reductions due to toxicity occurred in well over 50% of patient with both agents (27, 30). Most common AEs include hypertension, palmar-plantar erythrodysaesthesia syndrome, fatigue, weight loss, diarrhea, and stomatitis.

#### Cabozantinib

In September 2021, a third MKI, cabozantinib, was approved by the FDA as a second line therapy for patients with locally advanced or metastatic RR-DTC that has progressed following prior VEGF-R targeted therapy. Cabozantinib inhibits multiple tyrosine kinases involved in tumor growth and angiogenesis including VEGF-R2, AXL, c-MET and RET (5, 23). Notably, upregulation of c-MET and AXL signaling has been shown to play a role in resistance to antiangiogenic agents (31, 32), which serves as a premise for the use of cabozantinib in patients who have progressed on VEGF-R TKIs. Cabozantinib has been approved and widely used since 2012 for the treatment of advanced medullary thyroid carcinoma (MTC). More recent approval of this drug in DTC was based on results from COSMIC-311 (23), a double-blind, phase 3 placebo-controlled trial in which 258 patients with RR-DTC that had progressed on or following prior VEGF-R TKI treatment were randomized 2:1 to cabozantinib or placebo. Patients had to have received previous treatment with at least lenvatinib or sorafenib, and no more than two previous VEGF-R TKIs were allowed. Patients who progressed on placebo could crossover to open label cabozantinib. PFS was significantly prolonged in the group treated with cabozantinib (11.0 versus 1.9 months; HR 0.22, 96% CI 0.15-0.32; p < 0.0001) despite a short median follow-up of 10.1 months. PFS improvement was observed irrespective of previous treatment. ORR also favored cabozantinib (11% [95% CI 6.9%-16.9%] versus 0% [95% CI 0.0%-4.1%]; p=0.0003), with 18 confirmed partial responses (PR) in the treatment group as opposed to none in the placebo group. Overall, results of the COSMIC-311 trial were encouraging in a population of patients with aggressive disease that would have otherwise progressed rapidly, as illustrated by the very short median PFS in the placebo group, and in whom treatment options are limited.

Similar to sorafenib and lenvatinib, AEs are very frequent with cabozantinib, occurring in 94% of patients. Grade 3 or 4 AEs were observed in 57% of patients on cabozantinib, most frequently palmar-plantar erythrodysaesthesia syndrome, fatigue, hypertension, and diarrhea. These AEs were comparable to those reported in other studies and were manageable.

### FDA-approved selective kinase inhibitors

Although MKIs can significantly improve PFS in patients with advanced RR-DTCs, these therapies have multiple drawbacks. Their toxicity profile can have a major impact on patients’ quality of life and may limit their long-term effective use in clinical practice. For instance, real-life studies with lenvatinib describe treatment interruption and dose reduction rates as high as 79.5% (33). Moreover, as we will discuss below, many patients will eventually develop resistance to treatment and progress. For these reasons, the quest for treatments that do not target the angiogenic pathway and provide more personalized therapeutic options for patients with advanced DTCs has continued, culminating in the FDA-approval of various selective kinase inhibitors. These agents target more specifically one or a few kinases involved in tumorigenesis, which allows for better efficacy and most importantly less toxicity.

#### BRAF +/- MEK inhibitors

As mentioned earlier, the *BRAF* V600E mutation is the most frequent oncogenic driver in PTCs, present in 60% of cases, which makes it an attractive therapeutic target. BRAF + MEK inhibitors have been used for many years in other *BRAF*-mutated solid tumors, mainly melanoma and non-small cell lung carcinoma (NSCLC).

##### Dabrafenib and trametinib

The combination of dabrafenib and trametinib, a selective BRAF and MEK 1/2 inhibitor respectively, was FDA approved in 2018 for the treatment of locally advanced or metastatic *BRAF* V600E-mutant anaplastic thyroid cancer (ATC) and has significantly changed the treatment paradigm of these tumors which were previously viewed as a death sentence (34–37). Most recently, based on data from the ROAR (NCT02034110) (38) and NCI-MATCH (39) basket trials, the FDA granted in June 2022 an accelerated approval of dabrafenib in combination with trametinib for the treatment of patients with *BRAF* V600E-mutated metastatic or unresectable solid tumors who have progressed on prior treatment and have no other satisfactory treatment options, including thyroid cancers.

Dabrafenib was first shown to be promising in patients with DTC in a phase 1 basket trial (40). This led to a randomized, multicenter, open-label phase 2 trial in patients with *BRAF* mutated PTC (24). This study included 53 patients with progressive disease within 13 months before enrollment. Patients could have received up to three priors oral MKIs, excluding other selective BRAF or MEK inhibitors. Patients were randomized to dabrafenib monotherapy or dabrafenib in combination with trametinib, and primary endpoint was ORR in each group within the first 24 weeks of therapy. It was hypothesized that combination therapy would have superior clinical efficacy due to dual inhibition of the MAPK pathway as well as mitigation of potential mechanisms of resistance to dabrafenib through MEK kinase inhibition. Patients on dabrafenib alone were allowed to crossover to the combination group on disease progression. ORR, which included minor responses (defined as a 20 to 29% decrease in the sum of target lesions), was 42% (95% CI 23–63%) with dabrafenib and 48% (95% CI 29–68%) with dabrafenib + trametinib (p = 0.67). Median PFS was also not statistically different between the two groups (10.7 [CI 3.8-34.7] versus 15.1 [CI 12.3-37.3] months; p=0.65). The median OS was 37.9 months [CI 23.4–NR] with single-agent dabrafenib and 47.5 months [CI 27.9–57.8] with combination therapy (p = 0.99). Notably, of the 14 patients who crossed over at progression, 4 (29%) had an objective response, including 3 PRs and one minor response, and 8 had stable disease (SD). Grade 3 AEs occurred in about 50% of patients in both groups. Most frequent AEs associated with dabrafenib alone were skin disorders, fever, and hyperglycemia, while fever, hypophosphatemia and fatigue were most common with combination therapy. Skin disorders were strikingly less frequent with the combination compared to dabrafenib alone (33% VS 65% respectively). This trial, although not showing any superiority of combined BRAF and MEK inhibition over BRAF inhibitor therapy alone, did show prolonged PFS and OS with both treatment strategies, making dabrafenib +/- trametinib a therapeutic option for patients with advanced *BRAF*-mutated PTCs, especially when anti-angiogenic agents are contraindicated or associated with significant risk. This being said, there has been no direct comparisons between dabrafenib and MKIs such as lenvatinib in *BRAF*-mutated advanced DTCs to justify favoring one treatment over the other.

#### RET inhibitors

RET is a transmembrane glycoprotein receptor-tyrosine kinase (RTK). Ligand binding leads to RET homodimerization followed by trans-phosphorylation of tyrosine residues within the intracellular domains and activation of several signal transduction cascades involved in cellular proliferation, including the MAPK and PI3K pathways (41). Oncogenic activation of RET can occur through three main mechanisms: mutations leading to activation of the kinase domain by ligand-independent dimerization, mutations causing direct activation of the RET kinase domain, and chromosomal rearrangements producing chimeric proteins with constitutively active RET kinase domain (41, 42). Germline activating *RET* mutations are associated with multiple endocrine neoplasia type 2 (MEN2) syndromes, while somatic *RET* mutations are found in ~ 65% of all sporadic MTCs (41). *RET* rearrangements, on the other hand, have been identified in various solid tumors, including about 5 to 10% of PTCs, most frequently in children and in patients with prior exposure to radiation (41). *CCDC6-RET* and *NCOA4-RET* are the most frequently identified *RET* fusions in PTCs.

Involvement of *RET* alterations in tumorigenesis makes this RTK a potentially actionable therapeutic target. Moreover, tissue-specific *RET* knockout studies in mice, targeting the hematopoietic, neuronal, and lymphoid tissues, suggested that RET inhibition would most likely result in very little clinically significant AEs (43–45). This led to efforts aiming to identify selective RET inhibitors that would be used to treat *RET* mutated tumors in patients.

Selpercatinib (LOXO-292) and pralsetinib (BLU-667) are two potent and highly selective RET kinase inhibitors that have been recently FDA approved in 2020 for patients with *RET* fusion-positive DTCs and *RET*-mutant MTCs who require systemic therapy.

##### Selpercatinib

Efficacy of selpercatinib in *RET*-altered thyroid cancers was demonstrated in the LIBRETTO-001 trial (29). This phase 1/2 study included 19 patients with *RET* fusion-positive thyroid cancers, mainly PTCs (13/19). Most patients (79%) had had previous therapy with at least one MKI. In the *RET* fusion-positive DTC cohort, ORR was 79% (95% CI 54-94), including one CR and 14 PRs. Interestingly, 2/3 patients with poorly differentiated thyroid cancers (PDTC), 1/1 patient with OTC and 1/2 patients with ATC had PRs to therapy. Median PFS was not reached, but 64% of patients were progression-free at 1 year. Among all the patients with *RET*-altered thyroid cancers treated with selpercatinib in the trial (n=162), grade 3 or grade 4 treatment related AEs occurred in 28% and 2% respectively, most frequently hypertension (in 21% of patients) and increased cytolytic liver enzymes (increased alanine aminotransferase in 11% and asparte aminotransferase in 9%).

##### Pralsetinib

ARROW is a phase 1/2 trial evaluating the efficacy of pralsetinib in patients with *RET*-altered locally advanced or metastatic solid tumors, including thyroid carcinomas (46). Updated data presented at the 2022 American Society of Clinical Oncology (ASCO) meeting in 21 patients with previously treated *RET* fusion-positive thyroid cancers showed an ORR of 86% (95% CI 64-97), including 15 PRs. Duration of response was 17.5 months (95% CI 16.0 – NR) and PFS was 19.4 months (95% CI 13.0 – NR) (28). Similar to selpercatinib, pralsetinib was well tolerated, with a manageable safety profile. Most frequent grade 3 AEs were hypertension (17% of all trial patients) and cytopenia (neutropenia in 13%, lymphopenia in 11% and anaemia in 10%). One case of grade 5 pneumonia also occurred (46).

In both the LIBRETTO-001 and ARROW trials, AE-related dose reductions and treatment discontinuations were relatively low with only 2 and 4% of discontinuations of selpercatinib and pralsetinib respectively (28, 46).

#### NTRK inhibitors

The tropomyosin-receptor kinase (TRK) family of RTKs includes TRKA, TRKB and TRKC which are encoded respectively by the neurotrophic receptor tyrosine kinase genes *NTRK1*, *NTRK2* and *NTRK3 (* 47–49). Once activated, TRK RTKs signal through several downstream pathways involved in cellular proliferation, among which MAPK and PI3K/AKT. TRK receptors play an important role in the nervous system development (47). Oncogenic fusions leading to constitutive activation of the kinase domain have been described in all three *NTRK* genes, and these alterations have been identified in multiple solid tumors including colorectal cancer, lung cancer, and melanoma. In the thyroid, *NTRK*-driven malignancies are rare, found in 2-3% of thyroid cancers in adults, including PTCs, OTC, ATCs, and PDTCs (10, 48, 49). Like *RET*-fusions, *NTRK* fusions are more frequent in pediatric patients with PTCs (up to 25% of cases) as well as in patients with previous radiation exposure. Despite their rarity, *NTRK* fusion-positive thyroid cancers are important to identify as we have now two FDA-approved targeted TRK inhibitors which have demonstrated clinical safety and efficacy in patients with metastatic or unresectable solid tumors with *NTRK* gene fusion.

##### Larotrectinib

Larotrectinib is a highly selective and potent TRK inhibitor with central nervous system activity (CNS). A pooled analysis (26) of 28 patients with *NTRK* fusion-positive thyroid cancers treated with larotrectinib from three basket trials (50–52) showed an ORR of 71% (95% CI 51-87), including 2 CRs, 18 PRs and 4 SDs. All patients with CNS metastases at baseline had a PR. 24-month PFS and OS were respectively 69 and 76%. When excluding the 7 patients with ATC, ORR increased to 86% (95% CI 64-97) and 24-months PFS to 84%. Response to therapy was irrespective of previous systemic therapy: 13 patients with DTC who had one or more prior lines of systemic therapy had an ORR of 92%. Notably, AEs were mainly grade 1 and 2, with only two patients who experienced grade 3 treatment-related AEs (anaemia and lymphopenia). No patients required treatment discontinuation due to AEs and only 2 patients experienced AEs leading to dose reduction.

##### Entrectinib

Entrectinib is another potent TRK inhibitor which was specifically designed to have systemic activity and cross the blood-brain barrier (25, 53, 54). Entrectinib also exhibits inhibitory action against ALK and ROS1 tyrosine kinases, which have been involved in resistance to TKIs (5, 25, 53, 54). In April 2022, updated pooled data (25) from two phase 1 studies (ALKA-372-001 and STARTRK-1) (53) in patients with *NTRK, ROS1* or *ALK* alterations and one phase 2 basket study (STARTRK-2) (54) focusing on patients with *NTRK* fusion-positive solid tumors, were published. 13/121 patients had thyroid cancer, including 7 with CNS metastases. ORR was 53.8% (95% CI 25.1-80.8), median PFS was 19.9 months (95% CI 6.5-33.8), and OS was 19.9 months (14.5 – non evaluable [NE]). In the overall population, 11 patients had measurable CNS metastases at baseline, among which intracranial ORR was 63.6% (95%CI 30.8 – 89.1). Like larotrectinib, treatment related AEs were mostly grade 1/2, with AE-related treatment discontinuations in 8.3% of patients.

Therefore, larotrectinib and entrectinib appear as reliable and durable treatment options in *NTRK* fusion-positive thyroid cancers, including those with CNS metastases.

### Other non-FDA approved kinase inhibitors studied in DTC

We are frequently faced in clinical practice with patients that progress or do not tolerate the previously described FDA-approved treatments. In these situations, we resort to the off-label use of other anti-neoplastic agents that have been or are currently being studied in advanced DTCs and have shown some efficacy.

**Vemurafenib**, a selective BRAF inhibitor approved for treatment of *BRAF*-mutated melanoma, was in fact the first BRAF-inhibitor studied in DTC. Its efficacy was initially demonstrated in a small case series of 3 patients with metastatic *BRAF* V600E-mutated PTC (55). This was later confirmed by a phase 2 non-randomized, open-label, multicenter trial in which patients with recurrent or metastatic RAI-refractory *BRAF* V600E-mutated PTC, who were either TKI-naïve (cohort 1, n=26) or had progressed on VEGF-R TKI (cohort 2, n=22), received single-agent vemurafenib (56). In cohort 1, DCR with vemurafenib was 73% (95% CI 52-88) with 10 (38.5%) patients who had PR and 9 (35%) who had SD as best overall response. In cohort 2, response rates were lower, with 6 (27.3%) patients who had a PR as best overall response and 6 who had SD, leading to a DCR of 55% (95% CI 32-76). Median PFS was 18.2 months (95% CI 15.5-29.3) and 8.9 months (95% CI 5.5 -NE) in cohorts 1 and 2 respectively. Median OS was not yet reached in cohort 1, while it was 14.4 months (95% CI 8.2-29.5) in cohort 2. AEs were mostly grade 1-2, including rash, fatigue, alopecia, dysgeusia, creatinine increase and weight loss. Vemurafenib seems therefore to be a valid therapeutic option for *BRAF*-mutated PTCs, although it yet has to be studied in a phase 3 trial.

**Encorafenib** is another BRAF inhibitor, currently approved in combination with the MEK-inhibitor binimetinib for *BRAF*-mutated metastatic melanoma and colorectal carcinoma. It has a more than 10-times longer dissociation half-life than dabrafenib or vemurafenib, allowing more sustained target inhibition and potentially a more potent antitumor activity (57). Moreover, it is associated with low rates of pyrexia and photosensitivity which are the two main dose-limiting AEs with the dabrafenib/trametinib and vemurafenib/cobimetinib combinations, respectively (57, 58). Although no clinical data is currently available for its use in thyroid cancer, there is an ongoing phase 2 trial examining encorafenib combined with binimetinib, with or without immunotherapy (nivolumab), in patients with metastatic *BRAF* V600E mutant RR-DTC (NCT04061980). In practice, this drug can be considered as an alternative when dabrafenib is not tolerated, especially due to intractable fevers.

**Everolimus**, an inhibitor of mammalian target of rapamycin (mTOR), has been studied in several trials for treatment of advanced RR-DTCs. In fact, as previously discussed, activation of the PI3K/PTEN/AKT signaling pathway is frequent in advanced thyroid cancers. This is often due to a mutation of the PTEN protein, a PI3K inhibitor. Parallel activation of this pathway has also been suggested as an escape mechanism to TKIs. mTOR, a serine-threonine kinase, is a downstream effector of the PI3K/AKT pathway and serves as a potential therapeutic target. The first reported trial of everolimus in thyroid cancers was a multicenter, open-label, phase 2 study in South Korea that enrolled patients with all thyroid cancer histologies, including 6 patients with ATC and 9 with MTC (59). Among the 38 patients that were evaluable for response, DCR was 81%, including 2 PRs (both in DTC patients). 45% of patients showed durable SD for 24 weeks or longer. Median PFS in patients with DTC was 43 weeks. Treatment was overall well tolerated with mostly grade 1 AEs. This study was followed by a second phase 2 trial in the Netherlands, which enrolled 28 patients with advanced DTC, 54% of whom had previous treatment with a TKI, namely sorafenib (60). Sixty five percent of patients showed SD as their best response, with 58% having SD lasting more than 24 weeks. However, there were no PRs or CRs. Median PFS was 9 months (95% CI, 4-14), and median OS was 18 months (95% CI 7-29). Hanna and colleagues further expanded on the topic with another phase 2 trial, once again in all thyroid cancer histologies (61). In the DTC cohort (n=33), in which 51% of patients had previously been treated with a TKI, best response to therapy was SD in 82% and PR in 3%. Median PFS was 12.9 months (7.3-18.6), and median OS was not reached. Interestingly, in this trial, DTC patients with only a *BRAF* mutation had the longest PFS on everolimus, while patients with alterations in the PI3K/mTOR/AKT pathway did not show better response to therapy. Thus, these three phase 2 trials demonstrate that mTOR inhibition is a viable second-line option in patients who progress on TKI therapy.

The combination of **everolimus plus sorafenib** showed improvement of PFS in comparison with sorafenib alone in a randomized phase 2 trial in patients with RAI-R oncocytic thyroid carcinoma that included 34 evaluable patients (62). PFS was significantly improved in the sorafenib plus everolimus arm (24.7 months (95% CI 6.1-no upper) compared to the sorafenib arm (10.9 months (95% CI 5.5-no upper). Response rates were similar between groups.

**Pazopanib** is an antiangiogenic MKI that inhibits VEGF, FGF, PDGF, KIT and RET receptors. It is currently FDA approved for other solid tumors including renal cell carcinoma. Pazopanib was evaluated in two phase 2 trials looking at its efficacy in patients with RR-DTC (63, 64). In 2010, Bible and colleagues conducted a first trial in 37 patients, 18 of which had confirmed PR to therapy (response rate 49%; 95%CI 35-68). Responses were seen in 8/11 (73%) patients with follicular tumors, 5/11 (45%) patients with oncocytic tumors, and 5/15 (33%) patients with papillary tumors. Therapy was well tolerated with 46% of patients taking pazopanib for 12 months or longer. The most frequent AEs were fatigue, skin and hair hypopigmentation, diarrhea, and nausea. In 2020, the same group published a larger phase 2 international study in 60 patients with advanced or progressive RR-DTC treated with pazopanib. In this second trial, response rate was slightly lower, with 36.7% of patients having a PR (CI 24.6-50.1). This is probably explained by the fact that patients were more heavily pretreated than in the prior study (91.7% VS 27%). Median PFS was 11.4 months and median OS 2.6 years. Both studies did not show any differences in response to therapy between histological subtypes of DTC, nor according to mutation profile. There is therefore substantiating evidence to support the efficacy of pazopanib in RR-DTC, and it should be considered as a therapeutic option in patients who progress or do not tolerate other FDA-approved therapies.

Other kinase inhibitors, such as **sunitinib (** 65–67)**, vandetanib (** 68)**, axitinib (** 69–71) and **dovitinib (** 72) have been tested in thyroid cancer, all showing modest efficacy.

## Potential mechanisms of drug resistance

**Figure 2:**
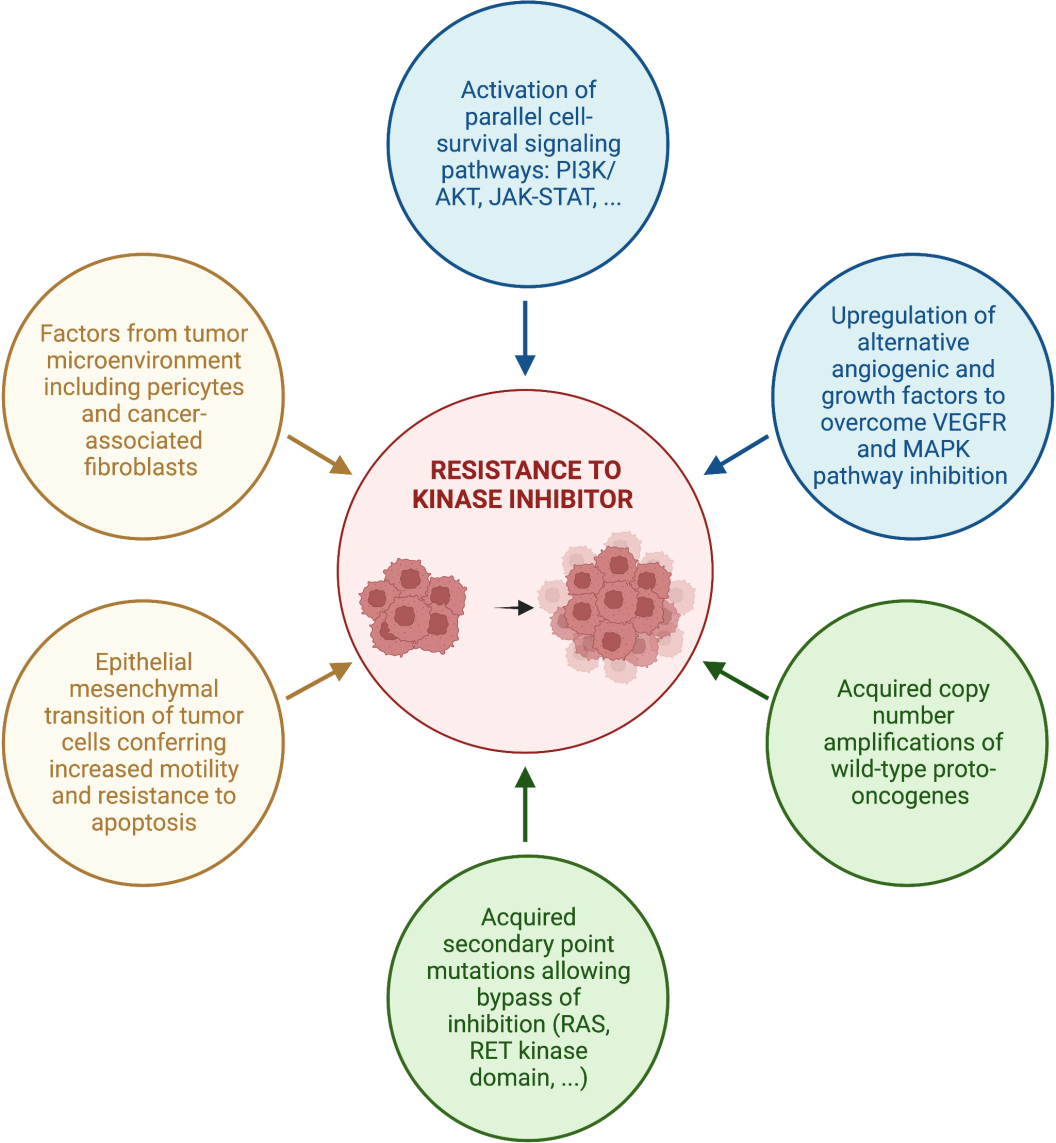
Proposed mechanisms of resistance to kinase inhibitors.

Despite promising initial results, all kinase inhibitors seem to become eventually ineffective, leading to inevitable disease progression. Current research efforts are therefore focused on identifying resistance mechanisms to kinase inhibitors and ways to overcome them. To date, a few potential mechanisms of tumor resistance to kinase inhibitors have been described (73) (**Figure 2**).

First, acquired resistance to tyrosine kinase inhibitors can involve escape mechanisms that activate parallel signaling pathways. For instance, upregulation of alternative angiogenic signaling factors such as FGF2, PDGF or epidermal growth factor receptor (EGFR) has been observed in tumors resistant to anti-VEGF TKIs (74–76). One possible factor underlying this phenomenon is hypoxia secondary to VEGF-R inhibition (74–76). In fact, hypoxia induces gene expression of proangiogenic factors primarily through the HIF-1α (hypoxia inducible factor-1α) transcription factor. Moreover, activation of the PI3K/AKT pathway or reactivation of the JAK-STAT pathway were also shown to be involved in acquired resistance to sorafenib (76). Similarly, several studies have demonstrated that cancer cells develop resistance to BRAF inhibitors by overexpressing growth factor receptors at their surface, including KIT, c-MET, EGFR and PDGF-receptor-β (PDFGR-β), leading to MAPK pathway reactivation despite BRAF inhibition (73, 77, 78). The treatment strategy to counteract this activation of alternate pathways is to either add a second TKI or to switch to another targeted systemic therapy. For example, in a multicenter phase 2 International Thyroid Oncology Group (ITOG) trial, cabozantinib conferred significant additional PFS and OS benefits (12.7 and 34.7 months respectively) in advanced DTC patients who had progressed on prior VEGF-R targeted therapy (79).

Factors associated to the tumor microenvironement have also been involved in resistance to kinase inhibitors. Pericytes are stromal cells that play a key role in the angiogenic microenvironement of thyroid cancers, in part by facilitating vessel maturation. PDGF growth factor-BB (PDGF-BB), which promotes pericyte proliferation through interaction with PDGFR-β, has been found to be increased in *BRAF* V600E-mutated PTCs, and pericytes have been shown to support the growth and survival of PTC cells (80, 81). Furthermore, *in vitro* studies suggest that pericytes might play a role in resistance to sorafenib and vemurafenib through secretion of thrombospondin-1 (TSP-1) and TGFβ1, which trigger rebound elevation in ERK1/2 and AKT levels allowing tumor cells to overcome inhibitory effects of these targeted therapies (82). Cancer-associated fibroblasts (CAF) have also been shown to promote cancer growth and to play a role in drug resistance (83).

Epithelial-mesenchymal transition (EMT) of tumor cells, induced by secondary mutations, hypoxia and other stimulating factors from the tumor microenvironment, was also shown to be involved in resistance to sorafenib (76) and lenvatinib (84). In fact, studies identified changes in treatment-resistant cells towards a mesenchymal morphology (76, 84, 85). Tumor cells undergoing EMT loose cell adhesion molecules such as E-cadherin and gain mesenchymal cell markers such as vimentin and N-cadherin, resulting in loss of cell-to-cell contacts and increased motility, which favor their dissemination to distant sites (84, 85). In addition, EMT makes tumor cells resistant to apoptosis and anti-tumor drugs (84, 85). Nonetheless, the exact interaction between EMT and anti-VEGFR TKIs resistance remains unknown.

Acquired wild-type copy number amplifications has also been identified as a resistance mechanism to BRAF inhibitors. For example, MCL1 copy number gain has been associated with resistance to vemurafenib treatment in PTC (86). MCL1 is an anti-apoptotic member of the BCL2 family, which might regulate parallel signaling pathways activating BRAF in PTCs resistant to anti-BRAF agents. Similarly, in another case report of a PTC which underwent ATC transformation while on dabrafenib (87), acquired triploidy of chromosome 7, which harbors the *EGFR, RAC1, MET*, and *BRAF* genes, was demonstrated in the progressive metastatic lesion. Copy number amplifications of these protooncogenes were consequently present in the dedifferentiated sample, probably contributing to tumor progression.

Finally, acquisition of secondary point mutations has also been proposed as a resistance mechanism to TKIs. For instance, a study exposing *BRAF* V600E mutated KTC1 thyroid cancer cells to long term vemurafenib showed development of secondary *KRAS* point mutations, allowing these cells to bypass BRAF inhibition (88). In addition to *RAS* point mutations, other acquired mutations that possibly confer drug resistance were found in the *RAC1, PTEN, NF1, NF2, TP53*, and *CDKN2A* genes (73, 87, 89). Moreover, it is now well recognized that acquired mutations in the RET kinase domain cause resistance to selective RET-inhibitors by interfering with drug binding. These include *RET* G810 solvent-front mutations as well as non-gatekeeper mutations at hinge (Y806C/N) and β2 strand (V738A) sites within the RET kinase domain (90–93).

Identification and better understanding of these resistance mechanisms pave the way for future novel therapies including combination of kinase inhibitors, potentiation of TKIs by adding immunotherapy, and redifferentiation therapy.

## Immunotherapy in DTC

In the past decade, immune checkpoint inhibitors (ICIs) have revolutionized cancer therapy. These monoclonal antibodies reactivate T-cell response against cancer cells, by blocking either the lymphocyte inhibitory receptor CTLA4 or the interaction between T-cell receptor PD-1 with its ligands PDL-1 and PDL-2 at the surface of cancer cells. To date, seven ICIs have received FDA approval for the treatment of various neoplasms including melanoma, NSCLC, renal cell carcinoma, and many others.

Just like in other neoplasia, thyroid cancer cells also escape immune surveillance, making ICIs an interesting therapeutic avenue. Immune escape in DTCs occurs through various mechanisms. First, deficient antigen presentation and reduced T-cell activation has been shown to play a role. This can occur either by downregulation of major histocompatibility complex (MHC) class I, mutations within the T-cell receptor binding domain of MHC-I, or loss of function of β2-microglobulin which results in disruption of MHC-I folding and transport to the cell surface (94, 95). Notably, MHC-I and β2-microglobulin expression were shown to be reduced or absent in 76% of PTCs (96).

Moreover, an immunosuppressive tumor microenvironment (TME) contributes to immune tolerance. Infiltration by regulatory T cells (Treg), which facilitate self-tolerance by suppressing effector T cells, has been observed in many tumor types (94). In PTC, increased Treg has been shown to correlate with lymph node metastasis and might be indicative of more aggressive disease (95, 97, 98). Other cells of the immune system including tumor-associated macrophages, plasmacytoid dendritic cells and tumor-associated mast cells are all overexpressed in the TME of DTCs and contribute to immune escape. Conversely, aberrant tumor vasculature that impairs the infiltration of immune cells can also occur (78). Finally, exhausted PD1^+^CD8^+^ T cells with defective cytokine production also play a role in the immunosuppressive milieu of DTCs (97, 98).

Several signaling pathways that are activated by oncogenic mutations associated with thyroid cancer can contribute to the immune escape. Among those, constitutive activation of the MAPK pathway impairs recruitment and function of tumor-infiltrating lymphocytes through increased expression of VEGF and multiple inhibitory cytokines (94, 95). Similarly, increased signaling in the PI3K pathway favors recruitment of inhibitory immune cells to the TME and reduces cytotoxic T-lymphocyte activity (94, 95).

Another major mechanism of immune escape in thyroid cancer as well as many other tumor types is up-regulation of inhibitory immune checkpoints, mainly PDL-1 but also PD-1, PDL-2 and CTLA4 (95). Notably, PDL-1 has been shown to be overexpressed in more advanced DTCs, with significant correlation between PDL-1 expression and lymph node metastasis, extrathyroidal invasion and disease-free survival (99). Interestingly, PDL-1 expression was higher in *BRAF* V600E mutant tumors, which are known to have the potential for more aggressive behavior.

Therefore, since pathogenesis of thyroid cancers includes escape of the immune system, reactivation of the anti-tumoral immune response may prove useful in the treatment of some thyroid neoplasia. This rationale led to various studies looking at ICIs in advanced DTCs.

### Pembrolizmab single agent

KEYNOTE-028 is a phase Ib clinical trial of the PD-1 targeting antibody pembrolizumab in patients with PDL-1 positive, locally advanced or metastatic DTC (100). Of note, patients did not need to have radioiodine refractory disease or progression to be enrolled in the study. 22 patients were treated with pembrolizumab 10mg/kg every 2 weeks for 24 months or until confirmed progressive disease, unacceptable AEs, or investigator or patient decision to withdraw. 50% of patients had previously received an MKI. ORR was 9% (95% CI 1-29%) with only 2 PRs. Clinical benefit rate, defined as PR + SD for at least 6 months, was of 50% (95% CI 28-72%). Median PFS was 7 months (95% CI 2-14 months). At data cutoff, median OS was not reached (95% CI, 22 months to not reached), with 6- and 12-month OS rates of 100 and 90%, respectively. Treatment was overall well tolerated, the most frequent AEs being diarrhea (in 32% of patients) and fatigue (in 18%). Only one grade 3 AE occurred, namely colitis, and no grade 4 AEs or AE-related treatment discontinuations were described.

### Lenvatinib and pembrolizumab combination

An ongoing phase 2 trial (NCT02973997) explores the combination of lenvatinib and pembrolizumab as a first line treatment of RR-DTCs with disease progression less than 14 month prior to enrollment (101).. In fact, VEGF has been associated with resistance to immune checkpoint blockade (94). VEGF axis promotes a hypoxic and immunosuppressive TME by decreasing T cell infiltration, impairing cytotoxic T cell activity, and promoting repressive immune cell infiltration. Thus, inhibition of VEGF signaling might represent an important strategy to enhance ICI efficacy. Inhibition of VEGF-R was correlated with improved response to ICIs in renal cell carcinoma, and the combination of lenvatinib + pembrolizumab has been approved for advanced endometrial carcinoma (102). Therefore, lenvatinib + pembrolizumab was also explored in DTC. Results reported in a poster at the 2020 ASCO meeting in 30 patients showed PR in 62% of patients and SD in 35%. Median time to tumor size nadir was 7.4 months (CI 1.6-17.8). Notably, 14/29 evaluable patients were still on therapy at data cutoff (7.6-18.9 months) and 6/19 (43%) patients had not yet reached tumor size nadir. Median PFS was not yet reached, but PFS at 12 months was 74%. Seventy percent of patients had grade 3 AEs and 10% had grade 4 AEs. The most common grade > 3 AEs were hypertension (in 47%), weight loss (in 13%) and maculopapular rash (in 13%). Therefore, the combination of lenvatinib + pembrolizumab seems promising, although it is unclear yet if addition of pembrolizumab brings any supplemental benefits to single agent lenvatinib as PR and SD rates with the combination are similar to those with lenvatinib alone (27). Updated data from the lenvatinib + pembrolizumab trial might help answer this question, especially if PFS or OS benefits are achieved.

### Cabozantinib and atezolizumab combination

Another ongoing multinational phase 1b trial, COSMIC-21 (NCT03170960), is evaluating cabozantinib in combination with the anti-PDL-1 antibody atezolizumab in advanced solid tumors, including DTCs. Similar to lenvatinib, cabozantinib has immunomodulatory properties that counteract tumor-induced immunosuppression and may enhance response to ICIs. Combination of cabozantinib and nivolumab, a PD-1 inhibitor, has already shown efficacy in a phase 3 randomized trial for advanced renal cell carcinoma (103). Efficacy and safety results of cabozantinib + atezolizumab as a first line therapy in 31 patients with locally advanced, metastatic and/or progressive RR-DTCs included in the COSMIC-021 trial were presented in a highlighted poster at the 2022 American Thyroid Association (ATA) meeting (104). Patients who had received any other systemic anticancer therapy were excluded. Fifty-eight percent of patients had PTC and 61% of the tumors were harboring a *BRAF* mutation. Patients were treated with cabozantinib 40mg daily and atezolizumab 1200mg every 3 weeks. At data cutoff, with a median follow-up of 24.9 months (95% CI 14.9-33.3), ORR was 42% (95% CI 25-61) including 13 PRs and 17 SDs. Impressively, DCR was 97% (30/31) with the remaining patient having no post-baseline assessment available. Duration of response to therapy was 22 months (95% CI 1.4 -28.0), median PFS was 15.2 months (95% CI 10.4-24.3), and 28/31 patients were still alive at data cutoff. Grade 3/4 AEs occurred in 55% of patients, mainly diarrhea (13%) and cytolytic transaminase increase (10%), with 4 patients having had to stop treatment due to AEs related to one or both drugs. Overall, AEs related to the combination therapy were consistent with those of the individual agents and were manageable. Therefore, first-line combination of cabozantinib + atezolizumab in advanced RR-DTC provided durable responses and a high rate of disease control across different subtypes of DTC, which makes it an interesting therapeutic option.

### Ipilimumab plus nivolumab

Combined CTLA4 and PD-1 blockade has also shown efficacy in multiple tumors including melanoma and renal cell carcinoma. In fact, combination therapy overcomes ICI resistance: CTLA4 inhibition increases T-cell priming and reduces Tregs in the TME, while PD-1 inhibition enhances T cell effector response (95). Preclinical data suggests that this combination could also be beneficial in aggressive RR-DTCs. Therefore, an ongoing phase 2 study (NCT03246958) is looking at the combination of nivolumab (an anti-PD-1) and ipilimumab (an anti-CTLA4) in RR-DTCs, including PDTCs (105). Results in 32 patients were presented in a poster at the 2020 ASCO meeting. Three (9%) patients achieved partial response, including one near-complete response, while 14/32 (44%) had stable disease. Median PFS at data cutoff was 4.9 months.

### Cabozantinib and ipilimumab plus nivolumab combination

Ipilimumab/nivolumab combination has also been studied in association with cabozantinib in a multicenter phase 2 trial looking at locally advanced or metastatic RR-DTCs that have progressed on one previous anti-VEGFR therapy (NCT03914300). Interim results in 11 patients were presented at the 2022 ATA meeting (106). Interestingly, 45% (5/11) of patients included in the study had OTC and 18% (2/11) had PDTC. ORR within the first 6 months, which was the trial’s primary endpoint, was 9% (1/11), while ORR at data cutoff was 18% with 6 SDs and 2 PRs. Median PFS and OS were respectively 9 months (3.0-NR) and 19.2 months (4.6-NR). Only 3 patients were still on trial treatment at data cutoff. Therefore, although this triple combination therapy was overall well tolerated, efficacy was very limited and ipilimumab+ nivolumab did not seem to offer any additional advantage to cabozantinib monotherapy.

Multiple other clinical trials looking at various ICIs in advanced DTC are currently underway, including a phase 2 trial studying encorafenib + binimetinib with or without nivolumab in patients with metastatic *BRAF* V600E mutant RR-DTC (NCT04061980), and a phase 2 trial evaluating the combination of the anti-PDL-1 durvalumab with the anti-CTLA4 tremelimumab in advanced RR-DTC (NCT03753919). **Table 2** summarizes ongoing and published trials of immunotherapy in DTC.

**Table 2:** Summary of ongoing and published trials of immunotherapy in DTC.

Drug	Trial	Number of patients	Study population	Efficacy results	Reference
**Atezolizumab +** **cabozantinib**	COSMIC-021 (NCT03170960), Phase Ib	31	Treatment-naïve, locally advanced, metastatic and/or progressive RR-DTCs	42% PR, 55% SD Median PFS: 15.2 months	Taylor et al. (104)
**Durvalumab + tremelimumab**	NCT03753919, phase II	N/A	Locally advanced or metastatic RR-DTC	Ongoing	N/A
**Ipilimumab+ nivolumab**	NCT03246958, Phase II	32	Metastatic RR-DTC with progression ≤ 13 months prior to enrollment	**Interim results:** 9% PR, 44% SD Median PFS: 4.9months	Lorch et al. (105)
**Ipilimumab/nivolumab + cabozantinib**	NCT03914300, Phase II	11	Locally advanced or metastatic RR-DTCs that have progressed on one previous anti-VEGFR therapy	**Interim results:** 18% PR, 54% SD Median PFS: 9 months	Konda et al. (106)
**Nivolumab +** **encorafenib/binimetinib**	NCT04061980, Phase II	N/A	Metastatic, *BRAF* V600E mutant RR-DTC	Ongoing	N/A
**Pembrolizumab** **(Single agent)**	KEYNOTE-028 (NCT02054806), Phase Ib	22	Locally advanced or metastatic DTC	PR+SD for at least 6 mos: 50% Median PFS: 7 months	Mehnert et al. (100)
**Pembrolizumab + lenvatinib**	NCT02973997, Phase II	30	Treatment-naïve, RR-DTC with progression ≤ 14 months prior to enrollment	62% PR, 35% SD Median PFS: NR 12-month PFS: 74%	Haugen et al. (101)

Mos, months; NR, not reached; N/A, not applicable.

## Redifferentiation therapy

Loss of RAI sensitivity in DTCs is associated with more aggressive disease and a significantly poorer prognosis. RAI refractoriness is due to loss of thyroid differentiation features, among which the most important is Na/I symporter (NIS) function and expression. In fact, NIS allows active iodine transport into follicular cells and is responsible for RAI entry into thyroid cancer cells. Immunohistochemistry studies have shown that NIS protein expression is significantly decreased in differentiated thyroid cancer tissues (107). Decreased targeting of NIS to the plasma membrane through reduced vesicular trafficking (108) and impaired cell-cell adhesion secondary to loss of E-cadherin (109) might also play a role in loss of RAI uptake in advanced thyroid cancers.

It has now been well demonstrated that MAPK pathway activation is associated with dedifferentiation and a decreased NIS expression (110, 111). Moreover, studies have shown that the degree of tumor dedifferentiation correlates with the magnitude of activation of the MAPK pathway, and that *BRAF* V600E mutations lead to greater MAPK activation than *RAS* or RTK alterations (11, 110, 111). Conversely, suppressing the MAPK pathway with BRAF or MEK inhibitors in mice was shown to restore NIS expression and RAI uptake (15). These findings opened the floor to redifferentiation therapy, a treatment strategy in which we aim to restore RAI uptake, allowing subsequent treatment with RAI in a tumor which was previously considered as RAI-refractory.

In the first clinical study looking at redifferentiation therapy, 24 patients with RR-DTC were treated with a MEK inhibitor, selumetinib, for 4 weeks (112). Of the 20 patients who could be evaluated, 60% (12/20) had increased uptake on subsequent ^124^I PET-CT scan, 8 of which reached the dosimetry threshold for radioiodine therapy (i.e. if one or more lesions could be treated with a dose of ≥ 2000 cGy with an ^131^I administered activity ≤ 300 mCi) and were therefore treated with RAI. During the 6 months-follow-up period after radioiodine therapy, a reduction in the size of target lesions was observed in all patients, with confirmed PR in 5/8 patients and SD in 3/8 as the best overall response. In the study cohort, 9 patients had tumors harboring a *BRAF* V600E mutation, 5 a *NRAS* mutation, 3 a *RET* fusion and 3 had no identified mutation.

Interestingly, in the selumetinib redifferentiation trial, the 8 patients who reached the dosimetry threshold included all 5 patients with an *NRAS* mutation but only 1 patient with a *BRAF* mutation (112). This led to the hypothesis that MEK inhibitors possibly achieve an incomplete blockade of MAPK signaling in *BRAF*-mutant tumors which harbor a higher degree of pathway activation. Therefore, this was followed by four trials evaluating redifferentiation with BRAF inhibitors in *BRAF*-mutated RR-DTC.

First, Rothenberg and colleagues (113) enrolled ten patients with *BRAF* V600E mutant RAI-refractory PTCs. Each patient received dabrafenib for 25 days, followed by an ^131^I whole body scan (WBS). 6/10 patients whose scan showed new sites of radioiodine uptake remained on dabrafenib for a total of 42 days, after which they received an empiric dose of 150 mCi of RAI. At 3 months, 2/6 patients had PR and 4/6 patients had SD.

Similarly, Dunn and colleagues (114) studied redifferentiation therapy using vemurafenib in a cohort of 12 patients with *BRAF* mutant RR-DTC, excluding OTCs. Patients were treated with vemurafenib for 4 weeks. Pre-treatment ^124^I PET-CT lesional dosimetry was done before and 4 weeks after vemurafenib therapy. Patients in whom at least one index tumor (of ≥ 5mm in maximal diameter) was predicted to absorb ≥2000 cGy with a clinically administered ^131^I activity of ≤ 300 mCi, identified as ^124^I responders, were subsequently treated with RAI while still on vemurafenib. 10/12 completed the 4-week treatment course of vemurafenib, and 4 of them were ^124^I responders, qualifying for RAI therapy. At 6 months, 2/4 patients had SD and 2/4 had a PR. Of these four patients, two required subsequent thyroid cancer treatment at 9 and 18 months, and the other two patients have not required further therapy at 22 and 33 months, suggesting prolonged benefits.

Weber and colleagues (115) performed a prospective phase II redifferentiation study in which 6 patients with *BRAF*-mutated RR-DTC were treated with dabrafenib + trametinib while 14 patients with *BRAF* wild-type tumors were treated with trametinib alone for 21 ± 3 days. Redifferentiation was achieved in 2/6 *BRAF*-mutated and 5/14 *BRAF* wild-type patients, all of which received a dosimetry-guided therapeutic dose of RAI. At one year, response to therapy per RECIST 1.1 was PR in 1/7 patient, SD in 5/7 patients and PD in 1/7 patient. Both *BRAF*-mutated patients had some decrease in tumor size following redifferentiation therapy (one PR and one SD).

Finally, Leboulleux et al. (116) recently published another prospective multicentric trial in which 21 patients with *BRAF*-mutated metastatic, progressive, RR-DTCs were treated with dabrafenib and trametinib for 42 days then received an empiric dose of RAI 150 mCi irrespective of uptake on diagnostic WBS. Only one patient had ^131^I uptake on baseline diagnostic WBS while 20 patients demonstrated uptake on the post-therapeutic WBS. Responses at six months were SD in 52% of patients, PR in 38% and PD in 10%, which corresponds to a tumor control rate of 90%. Eleven patients with PR at 6 or 12 months were re-treated with a second course of dabrafenib + trametinib followed by RAI. Nine of the 10 evaluable patients within this group had abnormal ^131^I uptake on the second post-treatment WBS. At 6 months, 6/10 patients had a PR and 1/10 a CR. The 12-month PFS rate was 82.0% (95% CI, 58.8-92.8). Notably, re-induction of ^131^I uptake and response rates following redifferentiation with dabrafenib and trametinib were higher in this study compared to what was reported by Weber et al. (115). Potential explanations for these differences include longer duration of drug therapy (42 *vs* 21 days), higher dose of dabrafenib (150 mg *vs* 75 mg twice daily), more limited tumor volume (no lesion larger than 3 cm) as well as empiric treatment of all patients regardless of restoration of uptake on diagnostic WBS in the trial by Leboulleux and colleagues.

Successful redifferentiation of *RAS* mutant tumors with MEK inhibition in the selumetinib pilot study (112) also led to a phase 2 trial looking at efficacy of the MEK 1/2 inhibitor trametinib for redifferentiation of *RAS* mutant and *RAS* wild-type RR-DTCs. 15/25 patients in the *RAS*-mutant cohort met the dosimetry threshold for radioiodine therapy on ^124^I PET, 14 of which received RAI. At 6 months, ORR was 32%, with 8 PRs (57%), 3 SDs (21%) and 2 PDs (21%). Six-month PFS in the *RAS* mutant patients was 44%. In the *RAS* wild-type cohort (n=9), 3/4 patients with *BRAF* Class II alterations and 1/4 patients with *RET* rearrangements qualified for RAI, with 3 SDs and 1 PR (in patient with a BRAF-altered tumor) (117).

Additional retrospective studies have confirmed that redifferentiation represents a promising new therapeutic approach in patients with advanced RR-DTCs. Jaber et al. (118) described 13 patients with RR-DTC in whom targeted therapy with either single-agent BRAF or MEK inhibitor, or combination of dabrafenib and trametinib (in one patient), led to increased ^131^I uptake. 9/13 patients were treated with RAI, all of whom had durable disease control (3 PRs, 6 SDs). Interestingly, *RAS*-mutated tumors seemed to have a better response to redifferentiation therapy compared to *BRAF*-mutated tumors in this study (118). On the other hand, Iravani et al (119) described 6 patients who received redifferentiation therapy with either trametinib in tumors harboring an *NRAS* mutation, or combined BRAF and MEK inhibition (with either dabrafenib+trametinib or vemurafenib + cobimetinib) in tumors with *BRAF* V600E mutations. Only 1/3 patients with an *NRAS* mutation but all 3 patients with a *BRAF* V600E mutation demonstrated restoration of RAI uptake and underwent subsequent RAI therapy. Of these 4 patients, 3 achieved PR and 1 had SD with a median follow-up of 16.6 months.

The concept of redifferentiation might also apply to tumors harboring other than *BRAF* or *RAS* mutations. For instance, Groussin and colleagues (120) described one case of successful redifferentiation therapy with Larotrectinib in a patient with metastatic PTC harboring an *EML4-NTRK3* gene fusion. Similarly, restoration of radioiodine uptake in patients with *RET*-fusion positive RR-DTC has been reported following treatment with selective RET-inhibitors pralsetinib (121) and selpercatinib (122).

Thus, substantial data now shows that mutation-guided MAPK pathway inhibition seems to be an efficient strategy to redifferentiate RR-DTCs (**Table 3**). However, available trials are significantly heterogeneous with regard to multiple aspects, including definition of radioiodine-refractory disease, inclusion criteria, duration of TKI therapy prior to RAI administration, choice of imaging modality to determine restoration of RAI uptake (^124^I PET/CT versus ^123^I scintigraphy) and dose of RAI (dosimetry-guided versus empiric). It also remains unclear whether increase of uptake on diagnostic WBS performed after treatment with the kinase inhibitors should be used as a criterion to select candidates for RAI administration. Therefore, more studies are needed to identify the optimal choice and duration of TKI before RAI, to better determine the characteristics of patients who are most likely to benefit from redifferentiation therapy, and to clarify the long-term risks as well as the duration of response to this therapeutic approach.

**Table 3:** Summary of redifferentiation therapy trials in DTC.

TKI	Duration of TKI prior to RAI	Evaluable patients (n)	Restoration of uptake (n)	Patients treated with RAI	Efficacy results	Reference
Selumetinib	4 weeks	20	12/20	5/5 *NRAS* MUT 1/9 *BRAF* MUT 1/3 *RET* fusion 1/3 WT	At 6 months: 5/8 PR 3/8 SD	Ho et al. (112)
Dabrafenib	6 weeks	10, all with *BRAF* V600E mutations	6/10	6	At 3 months: 2/6 PR 4/6 SD	Rothenberg et al. (113)
Vemurafenib	4 weeks	10, all with *BRAF* V600E mutations	6/10	4	At 3 months: 2/4 PR 2/4 SD	Dunn et al. (114)
Trametinib if *BRAF*-WT, dabrafenib + trametinib if *BRAF*-mutated	21 ± 3 days	20	7	5/14 *BRAF* WT 2/6 *BRAF* MUT	At 1 year: 1/7 PR 5/7 SD 1/7 PD	Weber et al. (115)
Dabrafenib + trametinib in *BRAF*-mutated RR-DTC	42 days	21	11/17 at 4 weeks 20/21 on PTWBS	21	At 6 months: 8/21 PR 11/21 SD 2/21 PD	Leboulleux et al. (116)
Trametinib	4 weeks	25 *RAS* MUT, 9 *RAS* WT	19	14/25 *RAS* MUT 3/4 *BRAF* MUT 1/4 *RET* altered	At 6 months: PR 9/18 SD 6/18 PD 3/18	Burman et al. (117)
BRAFi+/- MEKi if *BRAF*-mutated, MEKi if *RAS*-mutated, trametinib in WT patient	0.9 – 76.4 months	13	8/13	3/3 *RAS* MUT 5/9 *BRAF* MUT 1/1 WT*	3/9 PR 6/9 SD	Jaber el al (118).
Trametinib if *RAS*-mutated, dafrafenib/trametinib or vemurafenib/cobimetinib if BRAF-mutated	4 weeks	6	4/6	1/3 *RAS* MUT 3/3 *BRAF* MUT	3/4 PR 1/4 SD	Iravani et al. (119)

*Patient treated empirically with ^131^I despite no restoration of uptake on diagnostic whole-body scan.

WT, wild type; BRAFi, BRAF inhibitor; MEKi, MEK inhibitor; PTWBS, post-therapeutic whole body scan; MUT, mutant.

## Future perspectives in radioiodine refractory DTC

When tolerated, TKIs can lead to a significant decrease in tumor size and could allow surgical resection of a previously inoperable tumor: this is referred to as neoadjuvant chemotherapy. Most MKIs used in advanced DTC are anti-angiogenic and thus may lead to poor wound healing and fistula formation. Therefore, these drugs need to be discontinued several weeks before surgery, which makes them unfit for use in the neoadjuvant setting. Nevertheless, case reports have been published in which MKIs, mostly lenvatinib (123) and sorafenib (124), have been successfully used to achieve shrinkage of locally aggressive tumors invading major cervical vessels, allowing subsequent complete surgical resection. More recently, a systematic review of neoadjuvant targeted therapy in locally advanced thyroid cancer (125) reported an R0/R1 resection rate of 78.1% among 27 patients, across all thyroid cancer subtype including ATC, MTC and PDTC. This review included 18 patients with DTC, all of whom were treated with non-selective TKIs with anti-VEGFR activity (anlotinib, lenvatinib, sorafenib). Despite this, no increased hemorrhagic risk during surgery was reported. To further explore this therapeutic avenue, there is currently a phase II multicenter clinical trial examining the efficacy of neoadjuvant lenvatinib in patients with locally advanced DTC (NCT04321954).

Selective kinase inhibitors, on the other hand, have little to no anti-angiogenic properties, which makes them potentially safer in the neoadjuvant setting. A clinical trial looking at the neoadjuvant use of the selective BRAF-inhibitor vemurafenib in 17 patients with unresectable *BRAF*-mutated PTC, has been reported (126). Eleven patients who completed the 56 days of treatment with vemurafenib underwent subsequent surgery: 8 had a complete resection (R0), and 3 had a resection leaving only microscopic residual disease (R1). 3/11 patients had an incomplete resection. One patient, whose tumor was involving the carotid, had a fatal hemorrhage two weeks after surgery.

Although neoadjuvant use of targeted therapy is not standard in the management of locally advanced DTCs, this approach is promising and is being increasingly used in clinical practice, especially with the growing availability of specific kinase inhibitors. Nevertheless, more data is required to confirm the efficacy, safety, and long-term benefits of this treatment strategy. An ongoing trial looking at the use of neoadjuvant selpercatinib for locally advanced *RET*-altered thyroid cancers might help answer some of these concerns (NCT04759911).

Another major therapeutic avenue that is being explored for advanced radioiodine refractory thyroid carcinomas, resistant to existing treatments, is chimeric antigen receptor T-cell (CAR-T) therapy. CAR-T cells are genetically engineered T-cells that express a chimeric antigen receptor, which contains a single-chain variable fragment (scFv) responsible for antigen recognition, and an intracellular signaling domain which initiates T cell activation. CAR molecules can reprogram T-cells to recognize and eliminate tumor cells expressing specific antigens (127, 128). CAR-Ts have demonstrated remarkable efficacy in hematological neoplasms and are currently being studied in various solid tumors. However, use of CAR-T therapy is more challenging in solid tumors, due to an immunosuppressive tumor microenvironment that impedes the access of CAR-T cells into the tumor. Moreover, antigen selection in solid tumors can also be challenging, because many tumor antigens also have some low-level expression in normal tissues, exposing the patient to a risk of “on-target, off-tumor” toxicity (128). The TSH-receptor, a well-known thyroid specific antigen, seems to be a promising target for CAR-Ts in advanced DTCs in *in-vitro* and mouse models (129). Moreover, a study assessing the safety and tolerability of autologous CAR-T cells targeting intracellular adhesion molecular-1 (ICAM-1) in advanced refractory poorly differentiated thyroid cancers is currently ongoing (NCT04420754).

## Conclusion

Better understanding of the molecular mechanisms underlying thyroid cancer has revolutionized the treatment of advanced, radioiodine refractory disease. Over the past decade, we have seen an expansion in the use of kinase inhibitors for advanced thyroid cancers, with the most recent approval of six selective, less toxic, targeted agents. The increasing number of available drugs raises the question as to what is the optimal treatment sequence, which remains to be defined. Moreover, although these drugs offer a delay in disease progression and tumor size shrinkage, none have led to an improved length of survival. For many of these agents, drug related toxicity is non negligeable and can significantly alter quality of life. Furthermore, patients eventually develop resistance to these therapies and experience disease progression. Therefore, identification of the optimal timing for initiation of systemic therapy is crucial, taking into consideration disease burden and rate of progression, presence of symptoms, as well as patient comorbidities and toxicity profile of potential drugs. Given limitations of currently available therapies, the search for a curative treatment for RR-DTC, with long-term persistent efficacy, continues.

## Author contributions

SH: Conceptualization; Literature review; Data curation and Writing original draft. MC, M-CH, PI, MH and NB: Review and editing of original draft. RD: Conceptualization; Review and editing of original draft. All authors contributed to the article and approved the submitted version.
